# Exsection of Hip-Joint for Morbus Coxarius

**Published:** 1872-03-15

**Authors:** N. Senn

**Affiliations:** Ashford, Wis.


### EXSECTION OF HIP-JOINT FOR MORBUS COXARIUS.

BY N. SENN, M. D., ASHFORD, WIS.

L. C., æt. 10 years, the youngest member of a healthy family of children, was a vigorous, active boy until the latter part of summer in 1870. At that time he fell from a haystack, and as was thought at the time, sprained his hip. He was soon able to leave his bed, but walked quite lame. A few weeks after, the knee on the same side began to pain him, the hip became swollen and tender, violent constitutional symptoms followed, with hectic fever and rapid emaciation. In about three months an abscess formed in front of the upper part of the thigh, which soon opened spontaneously, and discharged a large quantity of pus.

The patient remained in a critical condition for several months, when he commenced to gain slowly, so that in the spring following he was able to walk on crutches.

During this time he had no medical treatment. When we saw him first, about six months ago, he was greatly emaciated; his countenance presented the appearance of severe and long-continued suffering. The affected limb was found in the following condition: Thigh strongly flexed and adducted; foot turned inward, so that the toes pointed towards the plantar arch of the opposite one; marked prominence of the great trochanter and gluteal region, with elevated gluteal crease. The limb was apparently greatly shortened, so that in the erect position only the tip of the toes would reach the floor; but on actual measurement this shortening was found to be owing mostly to the faulty position of the pelvis and thigh. Pressure over the great trochanter towards the pelvis, caused intense pain; there was no mobility in the joint. The limb was considerably smaller than its fellow on the opposite side.

The opening through which the abscess had first discharged had never healed, but was the outlet of a constant, profuse and debilitating discharge. It was situated on the anterior aspect of the thigh, at the inner border of the sartorius muscle, about eight inches below the anterior superior spinous process of the ilium. Through this opening a large probe could be passed up into the hip-joint, where dead bone was distinctly felt.

The general condition of the patient, the extensive destructive changes in the anatomical structures of the joint, and the conspicuous deformity, excluded every other means for the successful management of the case except the last alternative—exsection of the joint.

This operation we performed on the 11th September, 1871, assisted by Drs. Marston, of Newcassel; Loehr, of Theresa; and Lusck, of Mayville.

The method selected was a slight modification of M. Sentin's operation. After the patient was thoroughly under the influence of ether, a straight incision parallel to the femur was made, extending from two inches above the great trochanter to four inches below; another one, about two inches in extent, was made perpendicular to the first in the lower flap, from the joint downwards; the tissues around the trochanter, and the muscles attached to it, were now carefully separated from it with the knife, the posterior portion of the capsular ligament divided, when the femur was forcibly dislocated upwards and backwards by flexing the thigh strongly upon the pelvis, adducting and rotating it at the same time inwards. The greater portion of the head of the femur, with some smaller fragments, was found loose in the acetabulum. The bone was now divided just above the small trochanter with a chain saw. As the shaft of the femur along its inner side was found to be denuded of its periosteum, the incision downwards was enlarged, and another section, about four inches long, of the shaft removed; below this the bone and periosteum were healthy. The acetabulum was not affected. Nothing could be found of the ligamentum teres. The hemorrhage was very slight. The wound was washed out with a saturated aqueous solution of carbolic acid, the long incision closed by interrupted silver wire sutures and adhesive plaster; the short incision was kept open by inserting a tent into it, so as to afford a free outlet to the suppurative discharges that might follow afterwards. The whole wound was covered with lint saturated with the same solution—a compress and bandage to retain it in its position; a straight, long splint was placed on the outside of the limb, and confined by means of a roller.

No shock followed the operation; the patient came out from under the influence of the anæsthetic very kindly. On the third day we found him happy and cheerful; he had suffered but little pain since the operation; sleep and appetite good. The groin and thigh were considerably swollen; the large incision had united almost throughout its whole extent by the first intention; extension by means of weight and pulley was now applied, and the limb dressed as before. The wound was directed to be injected and dressed daily with carbolic acid oil (1–12).

From this time the patient was under the able treatment of Dr. Loehr. About three weeks after the operation the doctor applied a plaster Paris dressing, including the pelvis and the whole limb, with the exception of an opening over the wound, to give exit to the discharge.

This dressing more than satisfied our most sanguine expectations. It kept the limb perfectly immovable, which added materially to the comfort of the patient and his rapid improvement. In six weeks the wound had entirely healed, and has remained so since. Three months after the operation the plaster Paris dressing was removed, and a wire splint applied.

